# Characteristic Differences of Wind-Blown Sand Flow Field of Expressway Bridge and Subgrade and Their Implications on Expressway Design

**DOI:** 10.3390/s22113988

**Published:** 2022-05-24

**Authors:** Shengbo Xie, Xian Zhang, Yingjun Pang

**Affiliations:** 1 Key Laboratory of Desert and Desertification, Northwest Institute of Eco-Environment and Resources, Chinese Academy of Sciences, Lanzhou 730000, China; zhangxian@nieer.ac.cn; 2 University of Chinese Academy of Sciences, Beijing 100049, China; 3 Institute of Desertification Studies, Chinese Academy of Forestry, Beijing 100091, China; pangyingjun@caf.ac.cn

**Keywords:** expressway, bridge, subgrade, wind-blown sand flow field, sand transport

## Abstract

Bridges and subgrades are the main route forms for expressways. The ideal form for passing through sandy areas remains unclear. This study aims to understand the differences in the influence of expressway bridges and subgrades on the near-surface blown sand environment and movement laws, such as the difference in wind speed and profile around the bridge and subgrade, the difference in wind flow-field characteristics, and the difference in sand transport rate, to provide a scientific basis for the selection of expressway route forms in sandy areas. Therefore, a wind tunnel test was carried out by making models of a highway bridge and subgrade and comparing the environmental effects of wind sand on them. The disturbance in the bridge to near-surface blown sand activities was less than that of the subgrade. The variation ranges of the wind speed of the bridge and its upwind and downwind directions were lower than those of the subgrade. However, the required distance to recover the wind speed downwind of the bridge was greater than that of the subgrade, resulting in the sand transport rate of the bridge being lower than that of the subgrade. The variation in the wind field of the subgrade was more drastic than that of the bridge, but the required distance to recover the wind field downwind of the bridge was greater than that of the subgrade. In the wind speed-weakening area upwind, the wind speed-weakening range and intensity of the bridge were smaller than those of the subgrade. In the wind speed-increasing area on the top of the model, the wind speed-increasing range and intensity of the bridge were smaller than those of the subgrade. In the wind-speed-weakening area downwind, the wind speed weakening range of the bridge was greater than that of the subgrade, and the wind speed-weakening intensity was smaller than that of the subgrade. This investigation has theoretical and practical significance for the selection of expressway route forms in sandy areas.

## 1. Introduction

Blown sand is the main natural disaster that threatens road driving safety in sandy areas [1,2]. In such areas, blown sand is also an important factor in highway engineering wiring, survey and design, route form selection, construction, operation, and maintenance [3]. Expressways have become a symbol of modern traffic because of their advantages, such as large transportation volume, low cost, fast speed, high traffic efficiency, flexible mobility, few traffic accidents, and a high degree of intensive use of land resources. In recent years, with the development of the social economy, the improvement of the urbanization level, and the progress of automobile industry technology, expressways have developed rapidly and have become the primary land transportation mode in sandy areas. Compared with ordinary highways, the hazards of wind-blown sand expressways have the following characteristics: First, the vehicle speed is very high, and a slight sand accumulation on the road surface causes traffic accidents. Second, the isolation belt in the middle of the expressway easily becomes an obstacle to wind-sand flow. To avoid sand accumulation there controlling the wind-sand flow on the road is necessary. Third, because the minimum width of expressway pavement is 26 m, its thickness is approximately three times that of ordinary highways, making the environmental impact of such expressways considerable. In sandy areas in particular, the disturbance of the blown sand activity is more intense after construction. Furthermore, the near-surface wind speed, wind field, and sand transport rate are significantly changed, as are the erosion, transport and accumulation conditions of wind-blown sand flow [4,5]. Therefore, preventing and controlling the harm caused by wind-blown sand by relying on the road surface to transport sand is difficult. Highway lines have three basic forms: subgrades, bridges, and tunnels. Among them, tunnels are less affected by blown sand [6], whereas the effects of the blown sand environment on subgrades and bridges are obvious [7,8]. These scientific problems need to be solved urgently for the construction of expressways in sandy areas. Determining the route form that is reasonable for passing through sandy areas to reduce, or even avoid, the harm of wind-blown sand is necessary. At present, the relevant studies mainly focus on the wind-blown sand hazards of traffic engineering and blown sand environmental monitoring. These focuses include wind-induced fatigue and asymmetric damage in bridges [9], buffeting response analysis of bridges [10], wind-blown sand along railway infrastructures and mitigation measures thereof [11], remote measurement of aeolian sand transport on sandy beaches and dunes [12], satellite monitoring of dust storms [13], the law of sand particle accumulation over railway subgrade [14], estimation methods and techniques of aeolian sand transport rate [15], sand dune ridge alignment effects on the surface [16], damage by wind-blown sand and its control measures along desert highways [17], wireless wind data acquisition systems at arid coastal foredunes [18], and wind speed forecasting in traffic control decision support systems [19]. However, these studies focus on the form of a single highway and railway line, and systematic studies are lacking on the environmental effects of blown sand in the form of subgrades and bridges. In particular, the optimization selection of the form of expressway lines in sandy areas has not yet been reported. This investigation selected the subgrade and bridge forms for a comparative study of the environmental effects of blown sand to understand the differences in the influence of expressway bridges and subgrades on the near-surface blown sand environment and movement laws, such as the difference in wind speed and profile around the bridge and subgrade, the difference in wind flow-field characteristics, and the difference in sand transport rate. The contribution of this investigation is to provide technical support for the survey and design of expressways in sandy areas and for the selection of route forms.

## 2. Research Methods

### 2.1. Models and Their Dimensions

**Figure 1:**
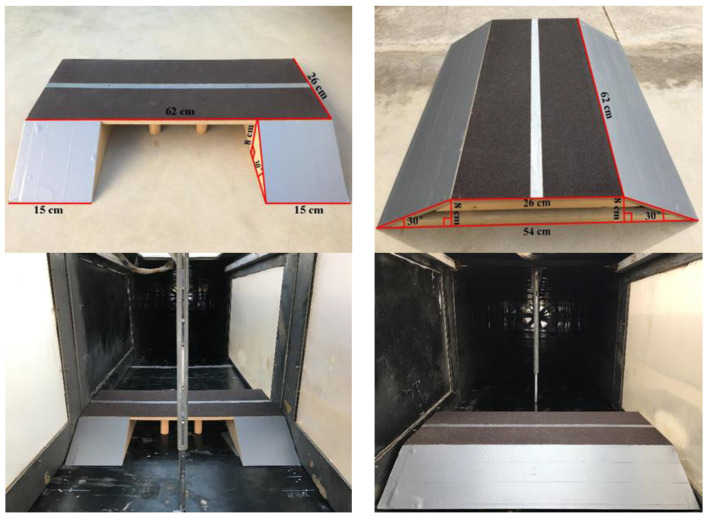
Photos of model size and wind tunnel test of expressway bridge and subgrade.

The expressway pavement width is 26 m and the slope ratio is 1:1.75, following expressway technical standards. The subgrade and bridge models of the wind tunnel test were constructed at a 1:100 scale, based on previous studies [20,21]. The wind tunnel used for the test had a cross-section of 63 cm × 63 cm and a boundary layer thickness of 12–15 cm. Therefore, the bridge and subgrade models were both 8 cm in height and 62 cm in length, and their blockage ratios were 7.1% and 12.5%, respectively. The model sizes are shown in Figure 1.

### 2.2. Layout of Wind Tunnel Test

#### 2.2.1. Layout of Wind Speed Test in Wind Tunnel

**Figure 2:**
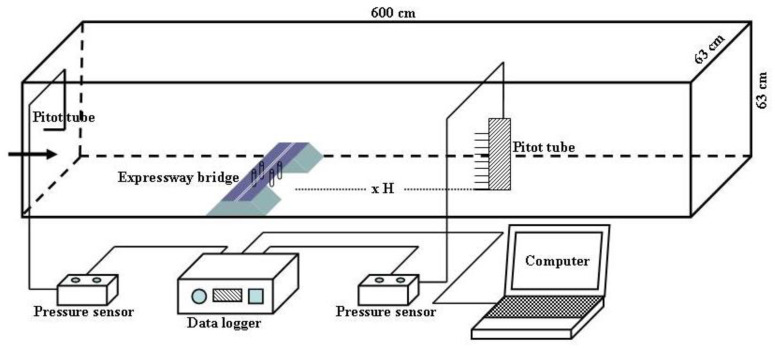
Wind speed experiment layout in wind tunnel.

**Figure 3:**
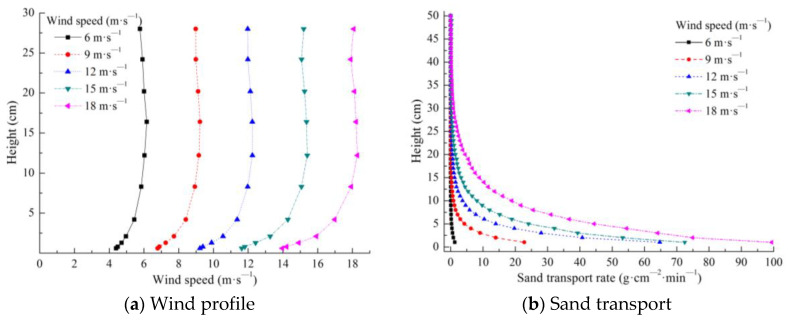
Wind profile and sand transport of wind tunnel test without model.

A total of 10 observation points were used: −30H −25H, −20H, −15H, −10H, −5H, −3H, −1H, −0.5H, and −0H upwind of the bridge (H represents the model height, − represents upwind, + represents downwind, and −0H represents the slope foot of the bridge windward side). The observation point settings upwind of the subgrade were identical to those of the bridge. Five observation points were set under the bridge: the bottom of the windward side slope middle, bottom of the windward side slope shoulder, bridge bottom center, bottom of the leeward side slope shoulder, and bottom of the leeward side slope middle. Five observation points were set on the surface of the subgrade: the slope middle of the windward side, slope shoulder of the windward side, subgrade top center, slope shoulder of the leeward side, and slope middle of the leeward side. A total of 12 observation points were used: 0H (representing the slope foot of the bridge leeward side), 0.5H, 1H, 3H, 5H, 10H, 15H, 20H, 25H, 30H, 35H, and 40H downwind of the bridge. The observation point settings downwind of the subgrade were the same as those of the bridge (Figure 2). The wind profile of the wind tunnel test without a model is shown in Figure 3a. In total, 10 different heights of wind speed at each position were converted using pitot tubes that were placed at the bottom center of the wind tunnel. The measured heights were 0.6, 0.8, 1.3, 2.1, 8.3, 12.2, 16.4, 20.2, 24.2, and 28 cm. The wind speed was measured every 2 s 30 consecutive times, and the average value was obtained. The test wind speeds were set in five groups: 6, 9, 12, 15, and 18 m·s^−1^.

#### 2.2.2. Test Layout of Sand Transport in Wind Tunnel

**Figure 4:**
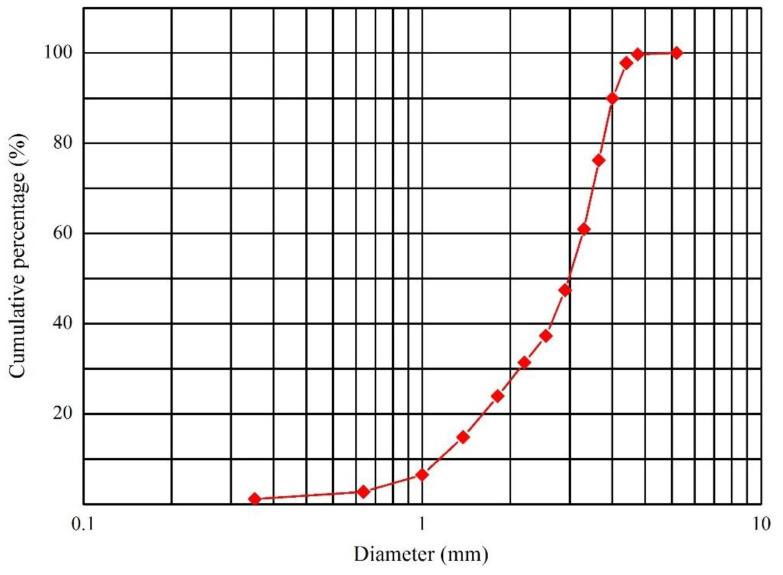
Grain size distribution curve of the test sand.

**Figure 5:**
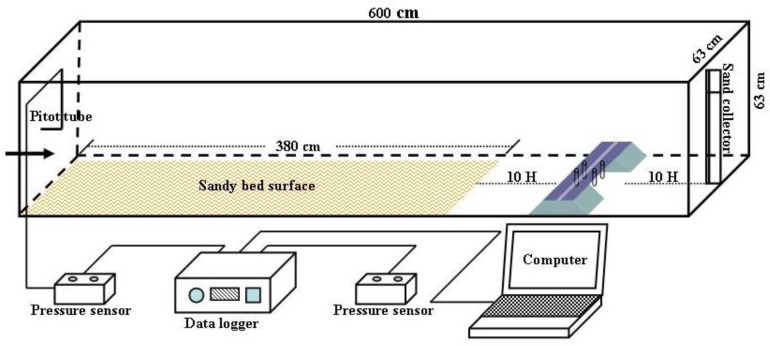
Test layout of sand transport in wind tunnel.

The sandy bed surface was laid at the 10H distance upwind of the bridge. The length, width, and thickness of the sandy bed surface were 380, 63, and 5 cm, respectively. The sand used in the experiment was obtained from the original surface sand of the Kumtag Desert, and the grain size distribution curve of the sand is shown in Figure 4. The sand collector was set at the 10H distance downwind of the bridge. Every 1 cm height set up a set of sand collector mouths (the width and height of the sand collector mouths were 2 and 1 cm, respectively), for a total of 50 heights. The sand-moving wind speed was 5.0 m·s^−1^. Therefore, the test wind speed was set to 6, 9, 12, 15, and 18 m·s^−1^, for five groups in total. The sand transport in the wind tunnel test without a model is shown in Figure 3b. The test layout of the wind tunnel used to measure the sand transport of the subgrade was the same as that of the bridge (Figure 5).

Simulation experiments in wind tunnels should generally satisfy the principles of geometric similarity, kinematic similarity, and dynamic similarity [22,23]. In this wind tunnel experiment, the sand used was taken from the original surface sand of the desert, conforming to geometric similarity. The bridge and subgrade are models made according to equal-scale reduction, also conforming to geometric similarity. The bridge and subgrade models were placed within the boundary layer of the wind tunnel test section, the pitot tubes were arranged at the bottom center of the wind tunnel test section, and the measured initial wind speed profile was consistent with the distribution law in nature (Figure 3a). Thus, this experiment conforms to kinematic similarity. In the model, the sandy bed surface was laid upwind, and the sand collector was set downwind. The measured initial sand transport rate was consistent with the distribution law in nature (Figure 3b), indicating that this experiment conforms to dynamic similarity. Therefore, this investigation satisfies the principles of geometric, kinematic, and dynamic similarity of wind tunnel simulation experiments to the greatest extent.

## 3. Test Results

### 3.1. Wind Speed at Each Observation Point

**Figure 6:**
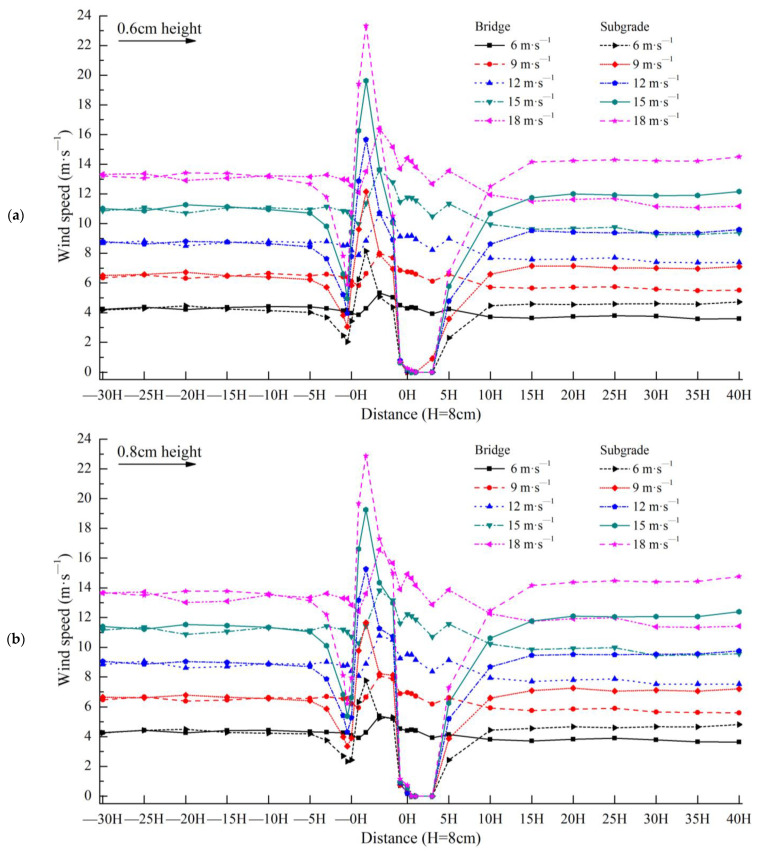

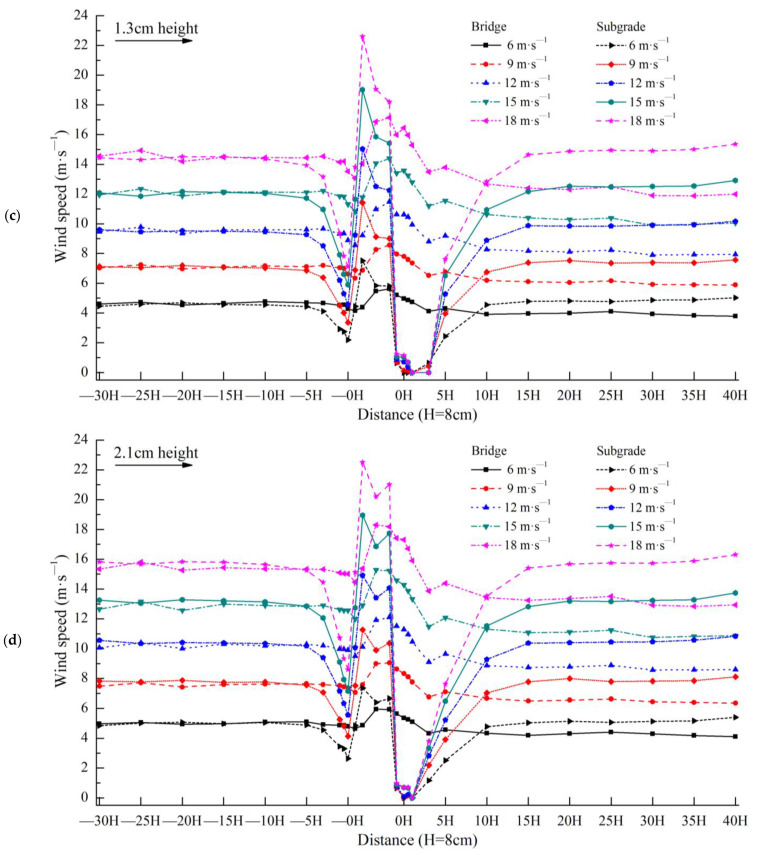

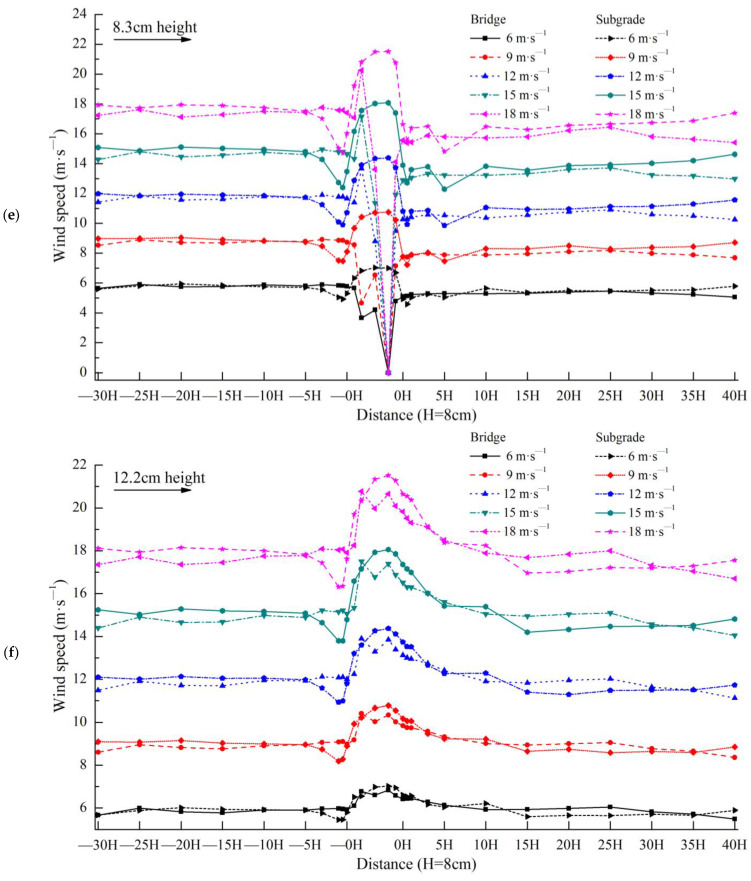

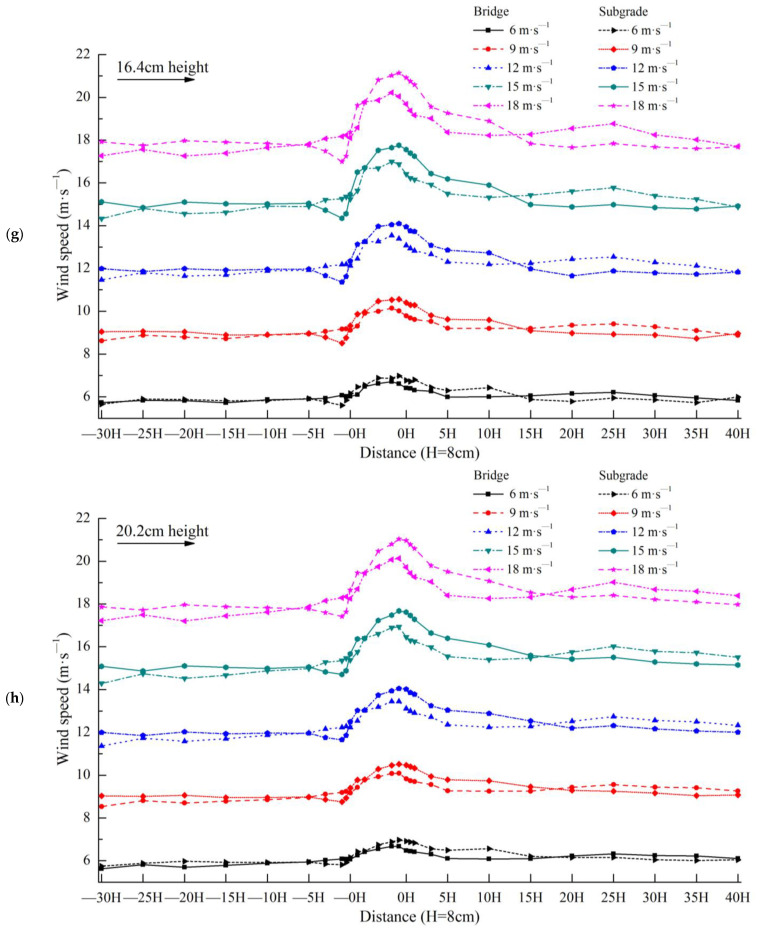

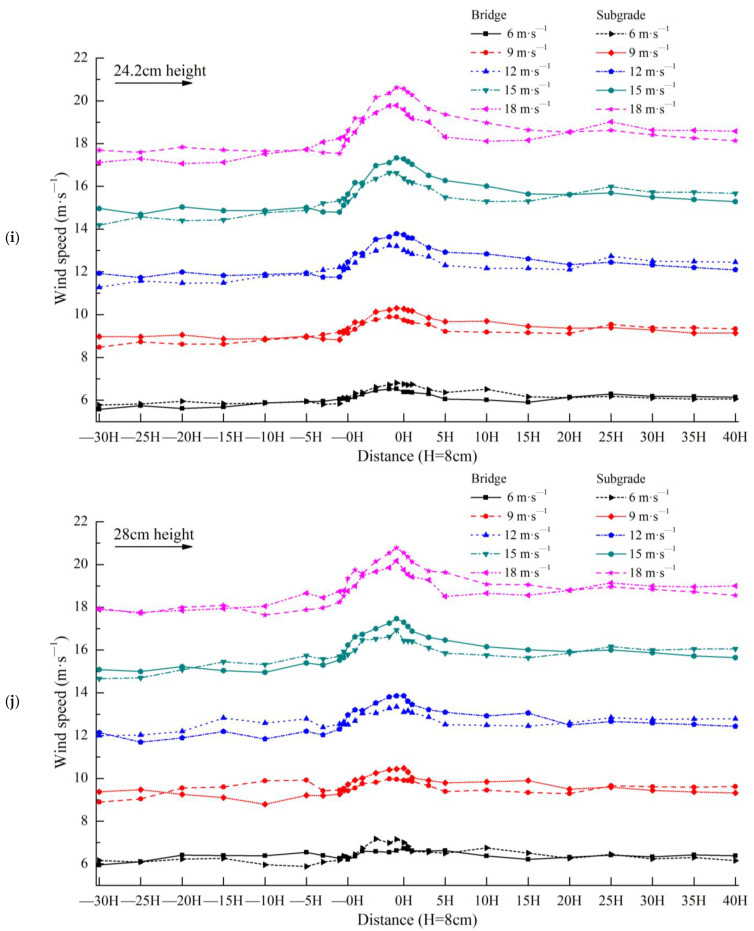
Wind speed differences between expressway bridge and subgrade.

According to the aforementioned layout of the wind speed experiment in a wind tunnel, the wind speed experiment results for the bridges and subgrades are shown in Figure 6.

When the height of the observation point was 0.6 cm (Figure 6a), the wind speeds of the bridge and the subgrade were nearly equal from −30H to −5H. From −5H to −0H, the wind speed of the bridge was significantly higher than that of the subgrade. From the slope middle of the windward side (the bridge is at the bottom of the windward side slope middle) to the center of the model (the bridge is the bottom center and the subgrade is the top center), the wind speed of the bridge was significantly lower than that of the subgrade. From the slope middle at the leeward side (the bridge is at the bottom of the leeward side slope middle) to 5H, the wind speed of the bridge was significantly higher than that of the subgrade. From 10H to 40H, the wind speed of the bridge was significantly lower than that of the subgrade. Notably, the wind speed of the subgrade recovered within this distance on the leeward side, but the wind speed of the bridge remained significantly lower than the corresponding wind speed on the windward side and did not recover. The variation range of bridge wind speed was significantly lower than that of the subgrade at the height of 0.6 cm.

When the height of the observation point was 0.8 cm (Figure 6b), the wind speed variations of the bridge and subgrade were very similar to those at 0.6 cm.

When the height of the observation point was 1.3 cm (Figure 6c), the wind speed variations of the bridge and subgrade were very similar to those at 0.6 cm.

When the height of the observation point was 2.1 cm (Figure 6d), the wind speed variations of the bridge and subgrade were very similar to those at 0.6 cm.

When the height of the observation point was 8.3 cm (Figure 6e), the wind speed of the bridge and that of the subgrade were nearly equal from −30H to −5H. From −3H to −0H, the wind speed of the bridge was significantly higher than that of the subgrade. From the slope middle of the windward side (the bridge is at the bottom of the windward side slope middle) to the slope middle of the leeward side (the bridge is at the bottom of the leeward side slope middle), the wind speed of the bridge is significantly lower than that of the subgrade. From 0H to 40H, the wind speed of the bridge and subgrade exhibited a minimal difference. The variation range of the bridge wind speed was greater than that of the subgrade at the height of 8.3 cm.

When the height of the observation point was 12.2 cm (Figure 6f), from −30H to −5H, the wind speed of the bridge and subgrade had a minimal overall difference. From −3H to −0H, the wind speed of the bridge was higher than that of the subgrade. From the center of the model (the bridge is the bottom center and the subgrade is the top center) to 1H, the wind speed of the bridge was lower than that of the subgrade. From 3H to 40H, the wind speed of the bridge and subgrade had minimal difference overall. The variation range of the bridge wind speed was lower than that of the subgrade at the height of 12.2 cm.

When the height of the observation point was 16.4 cm (Figure 6g), from −30H to −5H, the wind speed of the bridge and subgrade had a minimal overall difference. From −3H to −0.5H, the wind speed of the bridge is higher than that of the subgrade. From −0H to 10H, the wind speed of the bridge was lower than that of the subgrade. From 15H to 40H, the wind speed of the bridge and subgrade had minimal difference overall. The variation range of the bridge wind speed was lower than that of the subgrade at the height of 16.4 cm.

When the height of the observation point was 20.2 cm (Figure 6h), the wind speed variations in the bridge and subgrade were similar to those at 16.4 cm.

When the heights of the observation point were 24.2 and 28 cm (Figure 6i,j), although the wind speeds of the bridge and subgrade rose and fell with each other, they had a minimal difference overall. With the increase in height, the difference between the wind speeds of the bridge and subgrade decreased. The variations in their wind speeds became increasingly consistent, and the variation range of the wind speed became increasingly smaller. However, the variation range of the bridge wind speed was slightly lower than that of the subgrade wind speed.

### 3.2. Wind Flow Field

**Figure 7:**
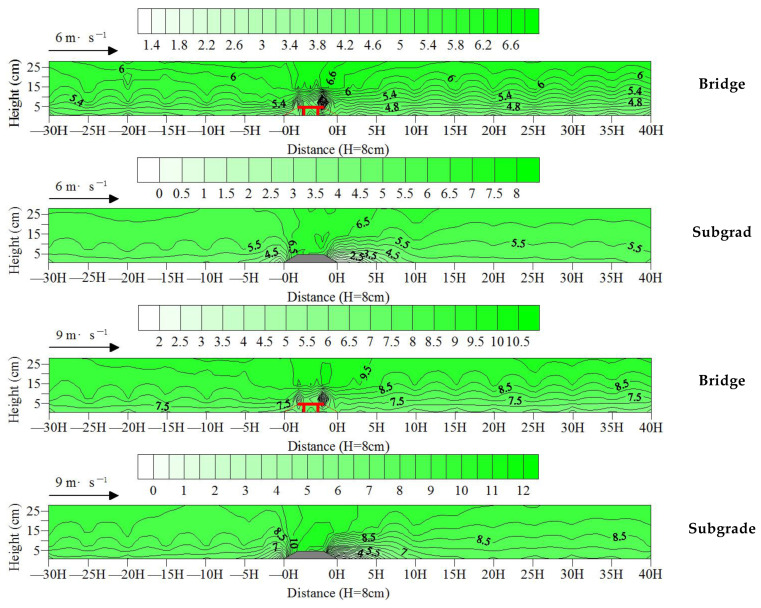

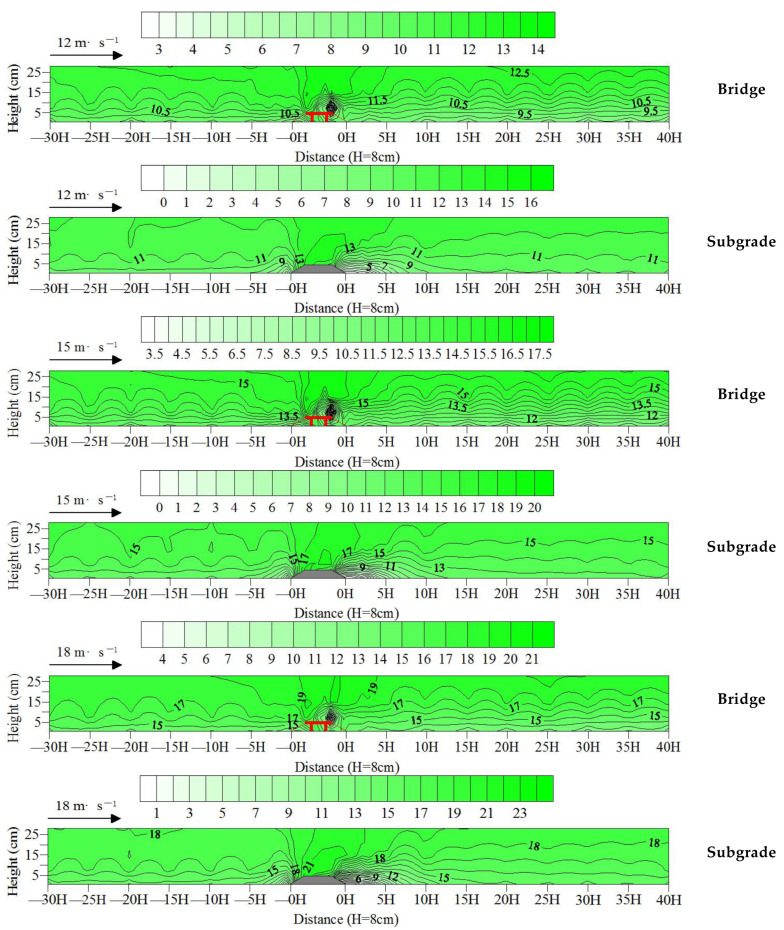
Wind field differences between expressway bridge and subgrade.

The Kriging interpolation method was used to draw the wind field map of the bridge and subgrade (wind speed contour map) based on the aforementioned wind speed test results. Figure 7 shows a wind-speed-weakening area between −3H upwind of the bridge to the slope shoulder of the windward side of the bridge. The figure also shows an obvious wind speed-weakening area between −5H upwind of the subgrade to the slope shoulder of the windward side of the subgrade. In these areas, the wind-speed-weakening range and intensity of the bridge are smaller than those of the subgrade. The wind-speed-increasing area was observed at the top of the bridge, as was an obvious increase in wind speed was at the top of the subgrade, particularly on the slope shoulder of the windward side. In those areas, the wind speed increase range and intensity of the bridge were smaller than those of the subgrade. A wind-speed-weakening area was found from the slope middle of the leeward side of the bridge to the 40H downwind, and an obvious wind-speed-weakening area was found from the slope shoulder of the leeward side of the subgrade to 15H downwind. In these areas, the wind-speed-weakening range of the bridge was greater than that of the subgrade, but the wind-speed-weakening intensity of the bridge was lower than that of the subgrade. These findings indicate that the overall range of wind speed variation of the bridge was lower than that of the subgrade, the wind field variation of the subgrade was more intense than that of the bridge, and the disturbance of the subgrade to the wind-blown sand environment was greater than that of the bridge.

### 3.3. Sand Transport

**Figure 8:**
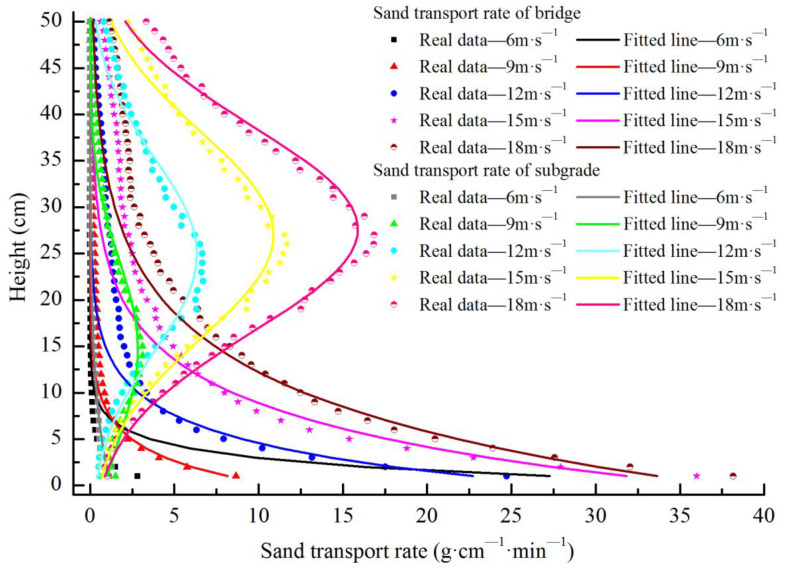
Sand transport rate of expressway bridge and subgrade.

The test results of the sand transport of bridges and subgrades are shown in Figure 8 and Table 1, based on the test layout of sand transport in the wind tunnel. The sand transport rates of the bridge for each height followed the distribution law of exponential functions, whereas the sand transport rates of the subgrade varying with height followed the distribution law of Gaussian functions. Under the experimental wind speeds of the five groups, the sand transport rates of the bridge were 7.56, 39.24, 141.05, 273.03, and 374.30 g·cm^−1^·min^−1^, and those of the subgrade were 9.56, 66.92, 164.16, 296.08, and 431.43 g·cm^−1^·min^−1^, respectively. The average sand transport rate of the bridge was 86.27% of that of the subgrade. The sand fluxes of the bridge were obviously lower than those of the subgrade, indicating that the passing rate of the wind-blown sand flow of the bridge was lower than that of the subgrade. Compared with the subgrade, the bridge blocks more wind-blown sand transport and causes more sand material to accumulate near the bridge.

## 4. Cause Analysis

In this test, the height of the model was set to 8 cm. Owing to the gap in the bridge, a minimal blocking effect was observed on the near-ground airflow below this height. When the airflow ran near the bridge, it passed through the gap at the bottom of the bridge. The disturbance received by the airflow was small. Therefore, the variation range of the wind speed was small, similar to the wind speed and flow field of a railway bridge [21]. Under the same experimental conditions, because no gap existed in the subgrade, it had a considerable blocking effect on the airflow near the ground. When the airflow ran in the −5H to −0H range upwind of the subgrade, it can only pass through the top of the subgrade with obstructions, resulting in considerable disturbance. Therefore, the wind speed decreased significantly. When the airflow ran to the windward side of the subgrade, the wind speed increased fleetingly with a climbing windward slope and reached the maximum value when it ran the shoulder of the windward slope. Thus, the wind speed increased significantly. When the airflow passed over the top of the subgrade, due to the terrain decrease of the leeward slope, the airflow dispersed rapidly and the wind speed dropped sharply. The wind speed dropped to the lowest value (0 m·s^−1^ or close to 0 m·s^−1^) within 0H–3H downwind of the subgrade, and the wind speed dropped significantly. Then, as the distance increased, the influence of the subgrade on the airflow weakened and the wind speed recovered quickly. Consequently, the wind speed of the subgrade varied sharply, and the variation range was significantly higher than that of the bridge. Above 8.3 cm, with the increase in height, the influence of the bridge and subgrade became smaller and the airflow was less disturbed. Therefore, the wind speed difference between the bridge and subgrade became smaller, the variation trend of the wind speed was the same, and the variation range decreased.

Relevant studies show that sand transported by the wind accumulates around any type of obstacle [24,25], and the decrease in near-surface wind speed easily causes sand material accumulation, while the increase in wind speed easily causes blown sand flow erosion [26,27,28,29]. In the wind-speed-weakening area upwind, because the wind-speed-weakening range and intensity of the bridge were smaller than those of the subgrade, the range and intensity of sand material accumulation upwind of the bridge were smaller than those of the subgrade. In the wind-speed-increasing area at the top of the model, because the wind-speed-increasing range and intensity of the bridge were smaller than those of the subgrade, the erosion range and intensity of the wind-blown sand flow on the top of the bridge were smaller than those of the subgrade. In the wind-speed-weakening area downwind, because the wind-speed-weakening range of the bridge was greater than that of the subgrade, and the wind-speed-weakening intensity was smaller than that of the subgrade, the sand material accumulation range downwind of the bridge was larger than that of the subgrade. However, the accumulation intensity was smaller than that of the subgrade.

Relevant studies have shown that the influence of bridges on wind sand movement decreases with increasing height [21] and has no influence on the wind sand movement of the near-surface after reaching the threshold height. However, the opposite is true for the subgrade: the higher the subgrade, the stronger the disturbance to the wind-blown sand activity of the near-surface [30,31]. In this experiment, although the wind speed variation range of the bridge was less than that of the subgrade, the wind speed near the surface (below the model height of 8 cm) still did not recover within 40H downwind of the bridge. This lack of recovery resulted in the weakening of the driving force of wind-blown sand flow transport, a decrease in the passing rate, and more sand materials being intercepted near the bridge. Therefore, the sand transport rate of the bridge was lower than that of the subgrade. At the same time, these findings indicate that the required distance to recover the near-surface wind speed and its flow field downwind of the bridge is greater than that of the subgrade, causing the sand material accumulation range to also be greater than that of the subgrade.

## 5. Results Discussion

A comparison of the characteristics of the wind-blown sand environment of the expressway bridge and subgrade is shown in Table 2 through the test results and analysis. The disturbance of the bridge to the wind-blown sand environment was less than that of the subgrade in seven indices: the variation ranges of the wind speed, variation ranges of the flow field, wind-speed-weakening range and intensity in the wind-speed-weakening area upwind, wind-speed-increasing range and intensity in the wind-speed-increasing area on the top, and wind-speed-weakening intensity in the wind-speed-weakening area downwind. However, the disturbance of the bridge to the wind-blown sand environment was greater than that of the subgrade in several indices, such as the required distances to recover the wind speed and its flow field downwind and the wind-speed-weakening range in the wind-speed-weakening area downwind, thereby decreasing the passing rate of the wind-blown sand flow of the bridge and increasing the sand material accumulation. Therefore, from the perspective of prevention and control of wind-blown sand hazards, the wind-blown sand environment of the bridge was generally better than that of the subgrade.

According to the experimental results and their analysis, the following implications for practical engineering applications can be obtained. When surveying and designing expressways in sandy areas, if the construction cost is not considered, expressways through seriously blown sand areas should generally use the bridge form. However, when the downwind direction is limited by terrain such as river valleys or other special factors [32], the space is narrow, and the distance is limited, expressways should use the subgrade form. The subgrade height should also be lowered, and the bridge height should be raised as much as possible to reduce or even avoid sand disasters. Future work can focus on content such as the threshold distance of the downwind direction where the subgrade form should be adopted, environmental effects of wind-blown sand of bridges with different sizes (different heights and widths), and the threshold height of the bridge, which can avoid sand disasters.

## 6. Conclusions and Implications

At a height below 8.3 cm near the surface, the variation ranges of the wind speed of the bridge and its upwind and downwind directions were lower than those of the subgrade. However, the required distance to recover the wind speed downwind of the bridge was greater than that of the subgrade, resulting in the sand transport rate of the bridge being lower than that of the subgrade. Under the experimental wind speeds of the five groups, the average sand fluxes of the bridge was 86.27% of that of the subgrade. Above 8.3 cm, the wind speed difference between the bridge and subgrade became smaller, the variation trend of the wind speed was the same, and the variation range decreased.

The variation in the wind field of the subgrade was more drastic than that of the bridge, but the required distance to recover the wind field downwind of the subgrade was smaller than that of the bridge. In the wind speed-weakening area upwind, the wind speed-weakening range and intensity of the bridge were smaller than those of the subgrade. In the wind-speed-increasing area on the top of the model, the wind-speed-increasing range and intensity of the bridge were smaller than those of the subgrade. In the wind-speed-weakening area downwind, the wind-speed-weakening range of the bridge was greater than that of the subgrade, and the wind-speed-weakening intensity was smaller than that of the subgrade. From the perspective of prevention and control of wind-blown sand hazards, the wind-blown sand environment of the bridge was generally better than that of the subgrade. Therefore, expressways through seriously blown sand areas should prioritize the use of the bridge form.

## Figures and Tables

**Table 1:** Fitting results of sand transport rate of expressway bridge and subgrade.

Route Forms	Wind Speed (m·s^−1^)	Fitting Function Type	Fitting Function Formula	a	b	c	R^2^
Bridge	6	Exponential	y = a × exp(b × x)	45.30	−0.51		0.99
Bridge	9	Exponential	y = a × exp(b × x)	10.98	−0.30		0.98
Bridge	12	Exponential	y = a × exp(b × x)	28.60	−0.23		0.96
Bridge	15	Exponential	y = a × exp(b × x)	36.87	−0.15		0.96
Bridge	18	Exponential	y = a × exp(b × x)	37.48	−0.11		0.98
Subgrade	6	Gaussian	y = a × exp(−((x − b)/c)^2^)	999.70	−118.30	45.69	0.94
Subgrade	9	Gaussian	y = a × exp(−((x − b)/c)^2^)	2.84	14.67	13.68	0.95
Subgrade	12	Gaussian	y = a × exp(−((x − b)/c)^2^)	6.34	24.88	14.65	0.95
Subgrade	15	Gaussian	y = a × exp(−((x − b)/c)^2^)	10.88	26.99	15.69	0.97
Subgrade	18	Gaussian	y = a × exp(−((x − b)/c)^2^)	15.89	27.64	15.68	0.98

**Table 2:** Comparison of characteristics of wind-blown sand environment of expressway bridge and subgrade.

Environmental Indexes of Blown Sand		Contrast	Advantage Item	Disadvantage Item
Wind speed	Variation range	bridge < subgrade	bridge	subgrade
	Required distance to recover the wind speed	bridge > subgrade	subgrade	bridge
Wind flow field	Variation range	bridge < subgrade	bridge	subgrade
	Required distance to recover the wind field	bridge > subgrade	subgrade	bridge
Wind-speed-weakening area upwind	Range	bridge < subgrade	bridge	subgrade
	Intensity	bridge < subgrade	bridge	subgrade
Wind-speed-increasing area on the top	Range	bridge < subgrade	bridge	subgrade
	Intensity	bridge < subgrade	bridge	subgrade
Wind-speed-weakening area downwind	Range	bridge > subgrade	subgrade	bridge
	Intensity	bridge < subgrade	bridge	subgrade
Passing rate of wind-blown sand flow (Average under the experimental wind speed of five groups)	Ratio (bridge/subgrade)	0.8627	subgrade	bridge

## Data Availability

Not applicable.
